# Two-year outcomes of the APOLLON observational study of intravitreal aflibercept monotherapy in France in patients with diabetic macular edema

**DOI:** 10.1038/s41598-022-22838-1

**Published:** 2022-10-29

**Authors:** Jean-François Korobelnik, Vincent Daien, Céline Faure, Ramin Tadayoni, Audrey Giocanti-Aurégan, Corinne Dot, Laurent Kodjikian, Pascale Massin, the APOLLON study investigators, Céline Faure, Ramin Tadayoni, Audrey Giocanti-Aurégan, Corinne Dot, Laurent Kodjikian, Pascale Massin, Samir Abada, Jad Akesbi, Isabelle Akninb, Nicolas Alfonsi, Sandrine Allieu, Carl Arndt, Karim Atmani, Stephanie Baillif, Xavier Benouaich, Mounir Benzerroug, Laurence Béral, Barham Bodaghi, Pierre Bonicel, Alexandre Bourhis, Guilhem Cartry, Frédéric Chiambaretta, Christophe Chiquet, Catherine Creuzot-Garcher, Adil Darugar, Flore De Bats, Marie-Noelle Delyfer, Michel Di Nolfo, Marcel Dominguez, Brice Dugas, Olivier Genevois, Jérôme Guyomarch, Jérémie Halfon, Ruxandra Hera, Olivier Jankowski, Valérie Klinger, Edouard Koch, Soumia Laib, Marie-Laure Le Lez, Olivier Lebreton, Amélie Lecleire-Collet, Caroline Marc, Victor Margescu, Martine Mauget-Faÿsse, Solange Milazzo, Anne-Lise Montcriol, Chaker Nefzaoui, Hassiba Oubraham, Paul Plavosin, Olivier Rebollo, Gilles Ribstein, Maud Righini, Pierre-Raphaël Rothschild, Franck Rumen, Boris Rysanek, Maher Saleh, Magali Sampo, Philippe Schauer, Sarah Scheer, Jean-Philippe Theron, Jennyfer Zerbib

**Affiliations:** 1 Service d’Ophtalmologie, Hôpital Pellegrin, CHU de Bordeaux, Place Amélie Raba Léon, 33000 Bordeaux, France; 2 INSERM, Bordeaux Population Health Research Center, UMR1219, Université de Bordeaux, Bordeaux, France; 3 Hôpital Gui De Chauliac, Montpellier, France; 4 INSERM, Université de Montpellier, Montpellier, France; 5 Hôpital Privé Saint Martin, Ramsay Générale de Santé, Caen, France; 6 Hôpital Lariboisière, Université de Paris, Hôpital I, AP-HP, Hôpital Fondation Rothschild, Paris, France; 7 Avicenne, AP-HP, Université Paris 13, DHU Vision Et Handicaps, Bobigny, France; 8 HIA Desgenettes, Lyon, France; 9 École du Val de Grâce, Paris, France; 10 Department of Ophthalmology, Croix-Rousse University Hospital, Hospices Civils de Lyon, Lyon, France; 11 UMR-CNRS 5510 Matéis, University of Lyon, Villeurbanne, France; 12 CUDC, Hôpital Lariboisière, Paris, France; 13 Present Address: Centre d’Ophtalmologie Paris, Breteuil, France; 14 Hôpital François Quesnay, Mantes-La-Jolie, France; 15 Hôpital Des Quinze-Vingts, Paris, France; 16 Institut Parisien d’Ophtalmologie, Paris, France; 17 Retina Cannes, Clinique Oxford, Cannes, France; 18 Hôpital Général Notre Dame de La Miséricorde, Ajaccio, France; 19 Centre d’Ophtalmologie, Clinique Beau-Soleil, Montpellier, France; 20 Hôpital Universitaire Robert Debré, Reims, France; 21 Groupe Hospitalier Paris Saint Joseph, Paris, France; 22 Centre Hospitalier Universitaire de Nice, Hôpital Pasteur 2, Université Côte d’Azur, Nice, France; 23 Clinique de L’Union, Saint-Jean, France; 24 Pôle Tassigny, Angers, France; 25 CHU Pointe-À-Pitre Abymes, University Hospital of Pointe-À-Pitre Abymes, Pointe-À-Pitre, France; 26 CHU Pitié Salpêtrière, Paris, France; 27 CHR d’Orléans, Orléans, France; 28 Institut Ophtalmologique Sourdille-Atlantique, Saint Herblain, France; 29 CH de Perpignan, Perpignan, France; 30 CHU Gabriel Montpied, Clermont Ferrand, France; 31 CHU de Grenoble-Alpes, Grenoble, France; 32 CHU Le Bocage, Dijon, France; 33 Pôle Oise Ophtalmologie, Chamant, France; 34 Centre Ophtalmologique Pôle Vision, Val-d’Ouest Clinics, Écully, France; 35 Groupe Hospitalier Pellegrin, CHU de Bordeaux, Bordeaux, France; 36 Centre VISIS, Perpignan, France; 37 Centre Rétine Galien, Bordeaux, France; 38 Polyclinique Grand Sud, Nimes, France; 39 Clinique Saint Hilaire, Rouen, France; 40 CHU de Martinique, Fort-de-France, France; 41 13 Place Gaston Paillhou, Tours, France; 42 Alpes Rétine, Montbonnot-Saint-Martin, France; 43 Centre Ophtalmologique du Val d’Oise (OPH95), Osny, France; 44 CH de Mulhouse, Malhouse, France; 45 CH de Versailles (André Mignot), Le Chesnay, France; 46 CH de Villeneuve-Saint-Georges, Villeneuve-Saint-Georges, France; 47 CHU Bretonneau, Tours, France; 48 CHU de Nantes, Nantes, France; 49 Clinique Mathilde, Rouen, France; 50 CH d’Avignon Henri Duffaut, Avignon, France; 51 Résidence Les, 3 Rivières, Mandelieu-La-Napoule, France; 52 Hôpital Fondation Adolphe De Rothschild, Paris, France; 53 Hôpital Universitaire d’Amiens-Picardie, Amiens, France; 54 CHU de Montpellier, Montpellier, France; 55 CH de Toulon, Toulon, France; 56 1 Rue Pougin de La Maisonneuve, Montargis, France; 57 CH de Blois, Blois, France; 58 Centre d’Ophtalmologie du Lez, Montferrier Sur Lez, France; 59 11 Rue Stractman, Belfort, France; 60 Hôpital Saint Joseph, Marseille, France; 61 Hôpital Hôtel-Dieu, Paris, France; 62 Visiopôle, Lagord, France; 63 CHU de Caen, Caen, France; 64 Hôpital Jean Minjoz, Besançon, France; 65 Hôpital de La Timone, Marseille, France; 66 Clinique Thiers, Bordeaux, France; 67 CHNO Des Quinze-Vingts, Paris, France; 68 Institut Ophtalmique Nord de France, Somain, France; 69 Nice Retina, Nice, France

**Keywords:** Retinal diseases, Medical research

## Abstract

APOLLON (NCT02924311) was a prospective observational study to evaluate the effectiveness of intravitreal aflibercept (IVT-AFL) treatment of diabetic macular edema (DME) over 24 months in routine clinical practice in France. The primary endpoint was mean change from baseline in best-corrected visual acuity (BCVA; Early Treatment Diabetic Retinopathy Study letters) by 12 months, and safety was monitored throughout the study. Of 402 patients enrolled across 61 participating clinics and hospitals in France, 168 patients were followed for at least 24 months and included in the effectiveness analyses (79 treatment-naïve and 89 previously treated). After 24 months of IVT-AFL treatment, the mean (± standard deviation [SD]) change in BCVA from baseline was + 6.5 (± 10.7) letters in treatment-naïve patients (p < 0.001) and + 1.6 (± 17.0) letters in previously treated patients (p = 0.415) from a baseline of 63.8 (± 13.6) and 60.5 (± 16.5) letters. The mean number of IVT-AFL treatments over 24 months was 11.3 (± 4.9) and 11.9 (± 4.7) for treatment-naïve and previously treated patients. This final analysis of the APOLLON study indicated that following 24 months of IVT-AFL treatment in routine clinical practice in France, treatment-naïve patients with DME achieved significant gains in visual acuity and previously treated patients maintained prior visual acuity gains.

Trial registration number: NCT02924311.

## Introduction

Diabetic retinopathy and a common complication thereof, namely diabetic macular edema (DME), are major causes of vision loss in the working-age population^1,2^. Given the rising prevalence of diabetes and increased life expectancy globally, this is cause for concern due to the high level of resources and surgical skills required to manage these patients^3^.

Vascular endothelial growth factor (VEGF) levels are increased in the eyes of patients with diabetic retinopathy^4^, and anti-VEGF treatments have emerged as a first-line therapy for DME^1,2^. Intravitreal aflibercept (IVT-AFL; Eylea®, Regeneron Pharmaceuticals, Inc.; Rensselaer, NY, USA) was approved for the treatment of visual impairment due to DME on the basis of two Phase 3 studies (VIVID and VISTA), which showed the clear superiority of IVT-AFL over laser therapy in terms of both visual and anatomic outcomes^5,6^. However, in routine clinical practice, the long-term persistence of patients on anti-VEGF therapy is poor^7^, and those patients who do continue therapy receive fewer anti-VEGF injections compared with those in randomized clinical trials^8^.

Although the efficacy of IVT-AFL in DME treatment has been demonstrated in several clinical and real-world studies^9–11^, real-world evidence from French patients is not yet available. In France, IVT-AFL treatment of patients with DME is provided in accordance with the local product label, which adheres to the European Medicines Agency Summary of Product Characteristics for IVT-AFL^12^. However, decisions regarding dosage, duration, treatment frequency, and follow-up assessments are at the discretion of the prescribing physician, and for this reason, IVT-AFL treatment patterns may vary markedly. Thus, real-world findings regarding IVT-AFL effectiveness, treatment patterns, and safety in other countries or regions may not be readily extrapolated to France. Long-term observational studies can provide healthcare providers with valuable insights into routine clinical practice and may inform the conversations that physicians have with their patients. APOLLON (NCT02924311) was a 24-month observational study to evaluate the long-term, real-world effectiveness and treatment patterns of IVT-AFL in patients with DME in routine clinical practice in France. The preliminary, 12-month results of this study showed that IVT-AFL treatment was associated with improvements in functional and anatomic outcomes in both treatment-naïve and previously treated patients^13^. Here, we report the final, 24-month analysis of the APOLLON findings, which provide a long-term perspective of IVT-AFL treatment effectiveness, patterns, and persistence in France.

## Methods

### Study design

APOLLON (NCT02924311) was a prospective, multi-center, observational study conducted between September 2016 and August 2019 across 61 ophthalmology centers at public and private clinics and hospitals across France. Based on a report from the Diabetic Retinopathy Clinical Research Network^14^, in which a standard deviation (SD) of ± 11 letters was observed in the mean visual acuity change of patients with DME treated with IVT-AFL, a sample size of 385 patients was calculated to be required. Therefore, it was planned to enroll at least 400 patients in APOLLON.

Patients were eligible for enrollment if they had been diagnosed with DME; were either treatment-naïve or had received previous treatment with laser, steroids, or an anti-VEGF agent other than IVT-AFL; were aged ≥ 18 years with type 1 or type 2 diabetes; and had a baseline visual acuity of < 20/40. All treatment decisions, including the decision to treat with IVT-AFL, were made at the discretion of the prescribing physician according to local practice, with the recommended treatment regimen being five initial monthly doses of 2 mg IVT-AFL followed by one injection every 2 months, and that after 12 months of treatment, the interval between doses is shortened or prolonged according to the results of visual and anatomic evaluations^12^.

The APOLLON study was conducted in accordance with the International Conference on Harmonization guidelines for Good Clinical Practice and the ethical principles of the 1964 Declaration of Helsinki and its later amendments or comparable ethical standards. The study protocol and informed consent forms were reviewed and approved by the French Consultative Committee on the Processing of Information in Health Research and by the French National Medical Council prior to any patient being enrolled into the study. All patients provided written informed consent for participation in this study.

### Study endpoints

The primary endpoint of APOLLON was change from baseline in best-corrected visual acuity (BCVA) at 12 months in the treatment-naïve and previously treated cohorts^13^. Here, we report findings on the secondary endpoints, which included the mean change in BCVA and central retinal thickness (CRT) between baseline and 24 months, the presence of visible fluid after 24 months, and the mean number of IVT-AFL injections over 24 months. BCVA was recorded using Early Treatment Diabetic Retinopathy Study (ETDRS) letters or Snellen chart results converted to approximate ETDRS letter scores. Safety was monitored throughout the study.

The safety analysis set (SAS) included all patients who received ≥ 1 injection of IVT-AFL. Treatment-emergent adverse events (TEAEs) were summarized using the Medical Dictionary for Regulatory Activities coding system. The full analysis set (FAS) included all patients who received ≥ 1 injection of IVT-AFL in the study eye and who had a BCVA evaluation available at baseline for the study eye.

The study eye was defined as the eye in which IVT-AFL treatment was initiated at the initial visit (baseline). If both eyes were treated at baseline, the study eye was considered to be the eye with worse visual acuity. All effectiveness analyses were performed for the study eye only, whereas the safety analysis included all eyes receiving IVT-AFL.

### Statistical analysis

All patient-based data required for the purposes of this study were collected at baseline, after each IVT-AFL treatment during the first 5 months, and at Months 6, 12, and 24 thereafter. The Month 6, Month 12, and Month 24 data comprised data assessed within the timeframe of 4.5–6.5 months, 11–13 months, and 23–25 months from the first IVT-AFL treatment, respectively. All variables were analyzed descriptively with the appropriate statistical methods: categorical variables by frequency tables (absolute and relative frequencies) and continuous variables by sample statistics (i.e., mean, SD, minimum, median, quartiles, and maximum). Continuous variables were described by absolute values and as the mean change from baseline per analysis time point, where applicable. The Student t-test was applied to compare the mean BCVA at baseline with the mean BCVA at Months 12 and 24. All statistical analyses were explorative and descriptive in nature, and the study did not aim to confirm or reject pre-defined hypotheses.

In contrast with the 12-month APOLLON analysis in which the last observation carried forward approach was used^13^, this 24-month analysis used the available data only (i.e., excluded the missing values) and then compared these results to those of a sensitivity analysis based on multiple imputation with the Markov chain Monte Carlo (MCMC) method, in which 10,000 iterations were used to impute missing values (i.e., where no BCVA or CRT assessment data were available at defined time points)^15^. Statistical analyses were performed with SAS version 9.2 or higher (SAS Institute, Inc.; Cary, NC, USA).

## Results

### Patient disposition and baseline characteristics

**Figure 1:**
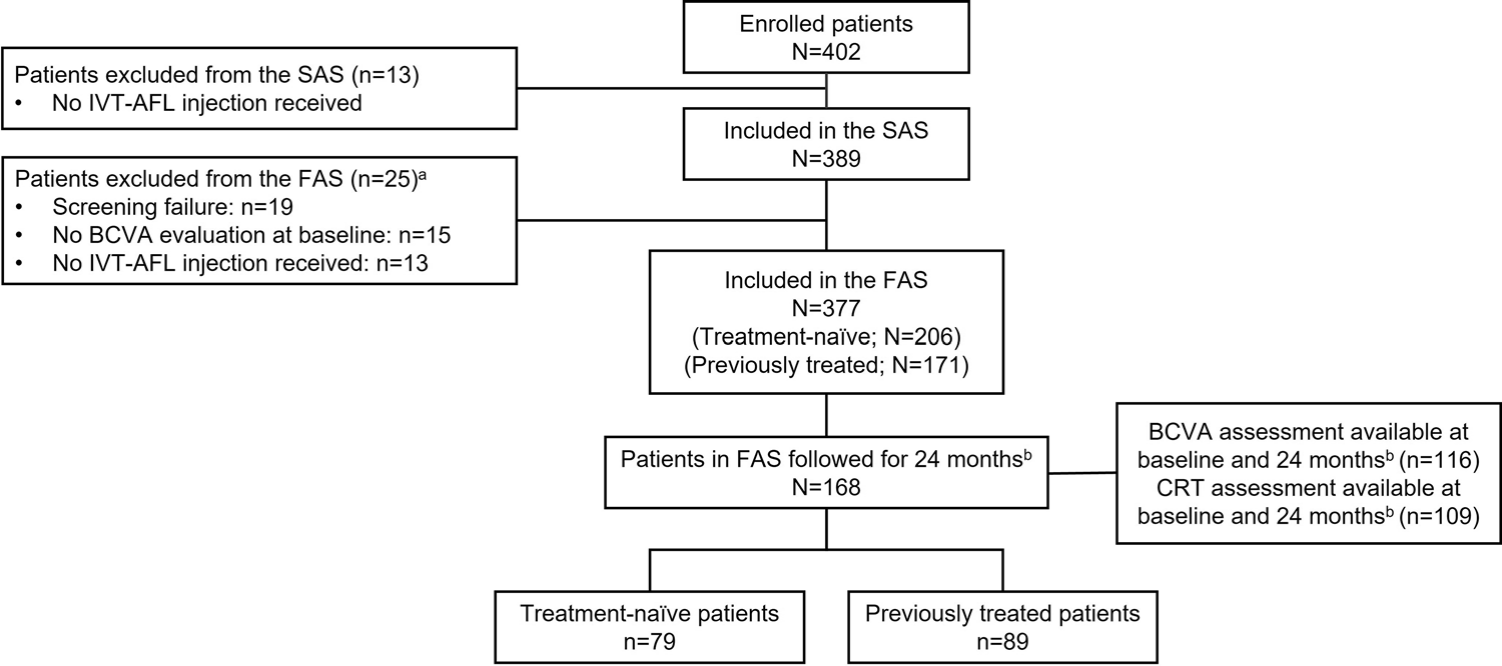
Patient disposition. BCVA, best-corrected visual acuity; CRT, central retinal thickness; FAS, full analysis set; IVT-AFL, intravitreal aflibercept; SAS, safety analysis set. ^a^Patients could have been excluded from the FAS for more than one reason. ^b^Patients had follow-up data available to at least 23 months, with a visit, injection, BCVA assessment, or optical coherence tomography evaluation between 23 and 25 months from the first IVT-AFL injection.

Of 402 patients enrolled, 13 did not receive IVT-AFL and were excluded from the SAS, whereas 25 patients were excluded from the FAS (Fig. 1). Of the 377 patients in the FAS, 338 patients were followed for ≥ 6 months, 290 patients were followed for ≥ 12 months, and 168 patients were followed for ≥ 24 months. Of the 168 patients followed for ≥ 24 months, 116 patients had BCVA assessments and 109 patients had CRT assessments available at both baseline and 24 months (i.e., between 23 and 25 months after the first IVT-AFL injection) (Fig. 1). The numbers of patients with BCVA and CRT data available at baseline, 6 months, 12 months, and 24 months are shown in Supplementary Fig. 1. The baseline demographics and disease characteristics of patients in the overall FAS and in the treatment-naïve and previously treated cohorts are shown in Table 1. For previously treated patients, the main reason for starting IVT-AFL treatment was the lack of efficacy of previous DME treatments (78.2%).

**Table 1:** Demographics and baseline characteristics of patients in the full analysis set.

	Overall FAS		
	Treatment-naïve (n = 206)	Previously treated (n = 171)	Total (N = 377)
Age, years	64.8 (± 12.0)	67.2 (± 9.8)	65.9 (± 11.1)
Male, n (%)	120 (58.3)	90 (52.6)	210 (55.7)
BMI, kg/m^**2**^	28.8 (± 6.2)	29.5 (± 5.4)	29.1 (± 5.8)
Prior treatment^**a**^			
Median time since last DME treatment, months (range)	–	9.8 (0–72)	–
Anti-VEGF agent, n (%)	–	107 (66.5)^b^	–
Ranibizumab, n (%)	–	102 (63.4)^b^	–
Photocoagulation laser, n (%)	–	107 (64.8)^c^	–
Intraocular steroids, n (%)	–	45 (27.3)^c^	–
Metabolic characteristics			
Systolic blood pressure, mmHg	141.6 (± 20.3)^d^	141.5 (± 17.9)^e^	141.5 (± 19)^f^
Diastolic blood pressure, mmHg	77.6 (± 12.5)^d^	78.0 (± 11.5)^g^	77.8 (± 12.0)^h^
HbA_1c_, %	7.8 (± 1.5)^i^	7.5 (± 1.3)^j^	7.7 (± 1.4)^k^
Type of diabetes, n (%)			
Type 1	30 (14.6)^l^	25 (14.7)^m^	55 (14.6)^n^
Type 2	176 (85.4)^l^	145 (85.3)^m^	321 (85.4)^n^
Median time since DME diagnosis, months (range)	1.2 (0–121)^o^	28.6 (0–258)^p^	6.4 (0–258)^q^
Visual and anatomic characteristics			
BCVA, ETDRS letters	60.8 (± 15.9)	58.6 (± 16.7)	59.8 (± 16.3)
< 24 letters, n (%)	8 (3.9)	7 (4.1)	15 (4.0)
24–70 letters, n (%)	139 (67.5)	125 (73.1)	264 (70.0)
> 70 letters, n (%)	59 (28.6)	39 (22.8)	98 (26.0)
CRT, μm	441 (± 123)^r^	453 (± 136)^c^	447 (± 129)^s^
SRF visible on OCT, n (%)	61 (31.3)^t^	37 (23.3)^u^	98 (27.7)^q^
IRF visible on OCT, n (%)	187 (95.4)^t^	156 (96.9)^u^	343 (96.1)^q^

*BCVA* best-corrected visual acuity; *BMI* body mass index; *CRT* central retinal thickness; *DME* diabetic macular edema; *ETDRS* Early Treatment Diabetic Retinopathy Study; *FAS* full analysis set; *HbA*_*1c*_, glycated hemoglobin; *IRF* intraretinal fluid; *OCT* optical coherence tomography; *SRF* subretinal fluid; *VEGF* vascular endothelial growth factor.

Values are mean (± standard deviation) unless otherwise stated.

^a^Patients may have received more than one previous treatment for DME.^b^n = 161; ^c^n = 165; ^d^n = 83; ^e^n = 76; ^f^n = 159; ^g^n = 74; ^h^n = 157; ^i^n = 124; ^j^n = 100; ^k^n = 224; ^l^n = 206; ^m^n = 170; ^n^n = 376; ^o^n = 204; ^p^n = 167; ^q^n = 371; ^r^n = 197; ^s^n = 362; ^t^n = 202; ^u^n = 169.

### Visual acuity at month 24

**Figure 2:**
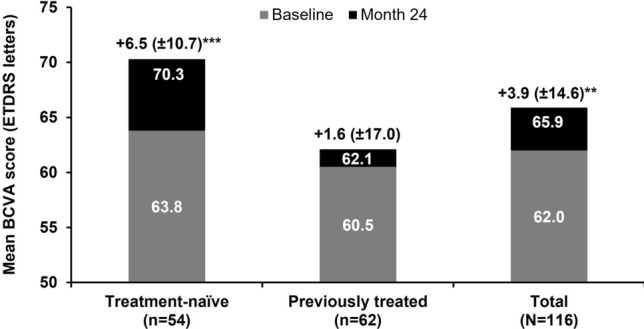
Change in visual acuity for all patients in the FAS with a BCVA assessment at baseline and Month 24, stratified according to treatment cohort. Values above each bar indicate the mean (± standard deviation) change in BCVA letter score from baseline to Month 24. BCVA, best-corrected visual acuity; ETDRS, Early Treatment Diabetic Retinopathy Study; FAS, full analysis set. ***p < 0.001 and **p < 0.01 for mean change at Month 24 versus baseline (Student t-test).

For patients from the FAS who had a BCVA assessment at both baseline and Month 24 (n = 116), the mean (± standard deviation [SD]) change in BCVA was + 6.5 (± 10.7) letters in the treatment-naïve cohort (baseline, 63.8 [± 13.6]; *p* < 0.001), + 1.6 (± 17.0) letters in the previously treated cohort (baseline, 60.5 [± 16.5]; not significant, *p* = 0.415), and + 3.9 (± 14.6) letters in the overall population (*p* < 0.01); see Fig. 2. A mean BCVA of ≥ 70 letters was achieved in 50.9% (59/116) of patients overall by Month 24, and in 61.1% (33/54) of treatment-naïve and 41.9% (26/62) of previously treated patients. A greater proportion of treatment-naïve patients experienced letter gains compared with previously treated patients (Supplementary Fig. 2); over 24 months, 6 patients overall (5.2%) lost 15 or more letters, whereas 22 patients (19.0%) gained 15 or more letters. As expected, patients with a higher BCVA at baseline reported the lowest change in BCVA at Month 24 (Supplementary Fig. 3). The sensitivity analysis (based on MCMC imputation of missing values) of the mean change in BCVA from baseline to Month 12 produced similar results (data not shown).

In the treatment-naïve patients from the FAS who were followed up for 24 months (n = 54), the mean (± SD) change in BCVA from baseline to Month 24 was + 5.6 (± 8.9) letters (95% confidence interval [CI]: 1.9, 9.2) in those who received five initial monthly doses of IVT-AFL (n = 25) and + 7.2 (± 12.1) letters (95% CI: 2.6, 11.8) in those who did not receive all five initial doses (n = 29).

### Anatomic outcomes at Month 24

**Figure 3:**
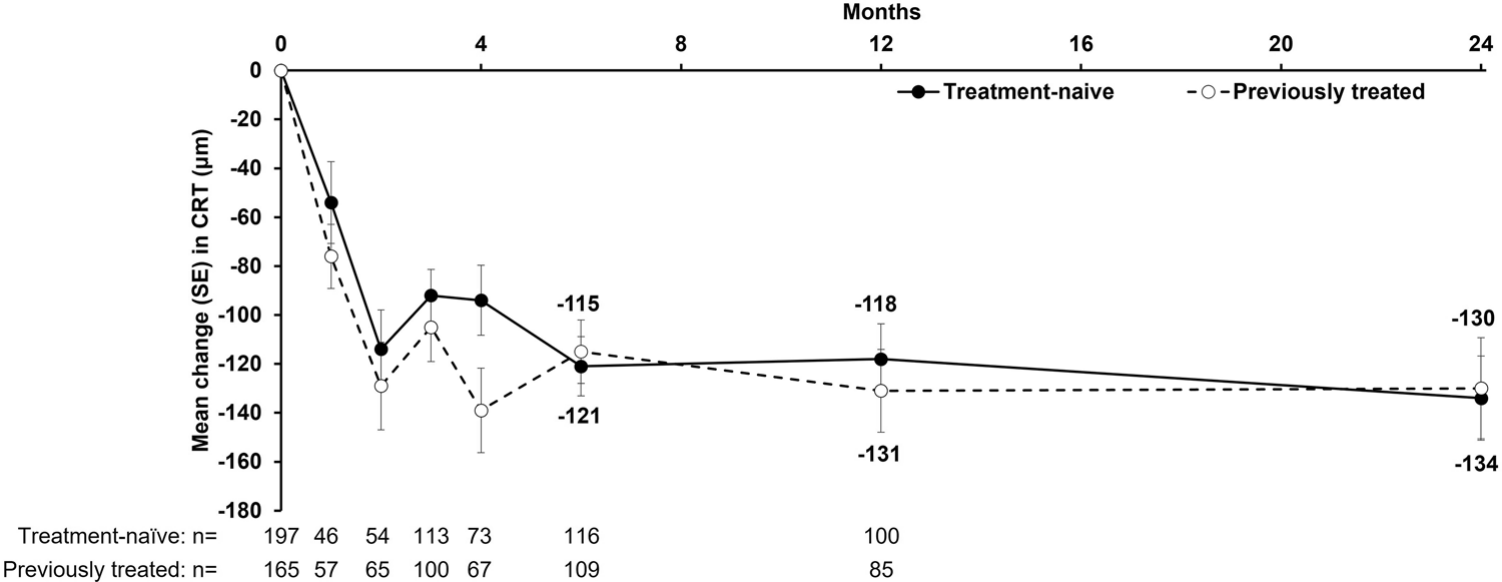
Mean CRT change over 24 months according to treatment cohort. Data are for all patients in the FAS with a CRT assessment at Month 24; error bars denote standard deviation. CRT, central retinal thickness; FAS, full analysis set.

For patients followed for 24 months, the mean (± SD) decrease in CRT was similar in the two treatment cohorts: from 446 (± 101) µm at baseline to 312 (± 82) µm at Month 24 in treatment-naïve patients (mean change: − 134 [± 123] µm; 95% CI: − 168, − 99), and from 442 (± 122) µm at baseline to 313 (± 104) µm at Month 24 in previously treated patients (mean change: − 130 [± 158] µm; 95% CI: − 171, − 88) (Fig. 3). The sensitivity analysis (based on MCMC imputation of missing values) of the mean change in CRT from baseline to Month 12 produced similar results (data not shown).

At baseline, subretinal fluid (SRF) was observed in 31.3% (61/195) of treatment-naïve and 23.3% (37/159) of previously treated patients who were assessed. At Months 12 and 24, the proportion of assessed patients with SRF decreased to 5.9% (6/102) and 7.0% (4/57) of treatment-naïve patients and 4.6% (4/87) and 5.4% (3/56) of previously treated patients. Baseline intraretinal fluid (IRF) was observed in > 95% of patients in both treatment cohorts, and at Months 12 and 24, the proportion of assessed patients with IRF decreased to 69.6% (71/102) and 68.4% (39/57) of treatment-naïve patients and 69.0% (60/87) and 69.1% (38/55) of previously treated patients. In the overall FAS, the change from baseline in the proportion of affected patients was − 27% for intraretinal fluid and − 22% for subretinal fluid by Month 24.

### Treatment exposure

**Figure 4:**
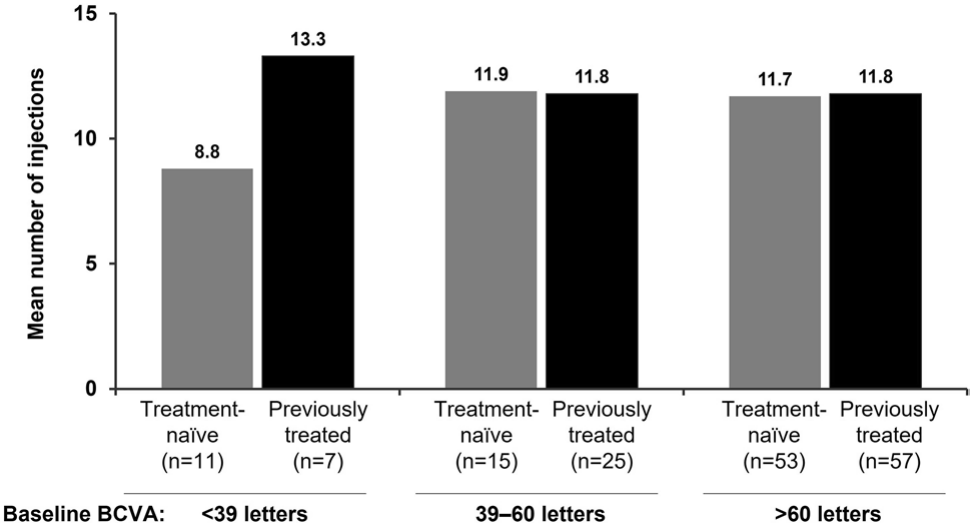
Number of injections received over 24 months according to baseline BCVA and treatment cohort in the overall FAS. Data are for all patients in the FAS with follow-up data on treatment exposure available to at least 23 months (i.e., with an injection between 23 and 25 months from the first IVT-AFL treatment). BCVA, best-corrected visual acuity; FAS, full analysis set; IVT-AFL, intravitreal aflibercept.

The mean (± SD) number of IVT-AFL injections over 24 months was 11.3 (± 4.9) for treatment-naïve and 11.9 (± 4.7) for previously treated patients (11.6 [± 4.8] for the overall FAS). The mean (± SD) number of follow-up visits per patient was 19.8 (± 5.3), and 82.7% of patients had ≥ 15 clinic visits during the 24-month observational period. The number of injections over 24 months was similar in patients with BCVA > 39 letters irrespective of prior treatment status but was numerically higher in previously treated patients with BCVA ≤ 39 letters and numerically lower in treatment-naïve patients with BCVA ≤ 39 letters (Fig. 4). The recommended five initial monthly doses of IVT-AFL were received by 40.8% of treatment-naïve patients, 34.5% of previously treated patients, and 37.9% of patients in the overall cohort. The mean (± SD) number of injections over 24 months in treatment-naïve patients was 12.6 (± 3.4) in those who received five initial monthly doses, and 11.1 (± 6.1) in those who did not receive all five initial doses.

In APOLLON, the two main reasons for early study discontinuation in the FAS (148/377 patients; 39.3%) were loss to follow-up (65/148; 44.2%) and switch to another treatment (57/148; 38.8%), and the main reason for switching treatment was lack of efficacy/response (32/57; 56.1%).

### Safety analysis at Month 24

Ocular TEAEs in the study eye occurred in 64.8% of patients in the SAS (Table 2); the most common event was cataract, which occurred in 8.5% of patients. Endophthalmitis occurred in one patient from each cohort. In one of these patients, endophthalmitis began 10 months after IVT-AFL treatment initiation and was considered serious and related to IVT-AFL, and IVT-AFL treatment was temporarily interrupted. Both cases of endophthalmitis were considered resolved/resolving at the end of the study. The second serious, treatment-related ocular TEAE reported was vitreous detachment, which occurred approximately 3 months after the first IVT-AFL treatment; this TEAE was resolved with sequelae and did not lead to any treatment interruption.

**Table 2:** Summary of the main safety events (SAS).

Patients, n (%)	Total N = 389
**Any ocular TEAE**	252 (64.8)
**Any ocular TEAE (> 2.0%)**	
Cataract	33 (8.5)
Diabetic retinal edema	25 (6.4)
Visual acuity reduced	16 (4.1)
Macular edema	15 (3.9)
Lacrimation increased	8 (2.1)
Ocular hypertension	8 (2.1)
Vitreous floaters	8 (2.1)
**Any serious ocular TEAE**	14 (3.6)
**Any serious non-ocular TEAE**	70 (18.0)
**Any serious treatment-related ocular TEAE**	2 (0.5)
**Any serious treatment-related non-ocular TEAE**	1 (0.3)
**Any ocular TEAE leading to discontinuation**	5 (1.3)
**Any non-ocular TEAE leading to discontinuation**	4 (1.0)
**Deaths**^a^	8 (2.1)
**Endophthalmitis**	2 (0.5)

^a^Assessed as unrelated to the study drug. SAS, safety analysis set; TEAE, treatment-emergent adverse event.

One patient experienced a serious, treatment-related, non-ocular TEAE, namely coronary artery stenosis, approximately 1 month after the first IVT-AFL treatment; this TEAE was resolved 7 days later without any interruption to IVT-AFL treatment. Eight (2.1%) patients died during the study, but none of the TEAEs leading to death were considered by the attending investigator to be related to the study drug or IVT-AFL injection procedure. The majority (6/8) of these deaths were due to cardiac disorders (including myocardial infarction, congestive cardiac failure, and cardiogenic shock), with the other two being due to renal and urinary disorders (renal failure and chronic kidney disease).

## Discussion

In France, IVT-AFL treatment of DME is fully reimbursed and prescribed in accordance with the European Medicines Agency Summary of Product Characteristics, which recommends five initial consecutive monthly injections, followed by one injection every 2 months^12^. After 12 months of treatment, the intervals between injections can be shortened or prolonged based on the outcomes of visual and anatomic assessments. According to the guidelines of the Haute Autorité de Santé (HAS; French Health Authority), only patients with a baseline visual acuity of < 20/40 are eligible for anti-VEGF treatment.

Although the efficacy of IVT-AFL in DME treatment has previously been demonstrated in several clinical and real-world studies^9–11^, no real-world evidence has yet been obtained in France, and the HAS requested the marketing authorization holder to perform further analyses regarding the use of IVT-AFL in routine clinical practice. In particular, the HAS requested information regarding the treated population, the conditions of use of the product, factors affecting persistence, and long-term evaluation of its effectiveness and safety. In response to the HAS requirements, APOLLON was conducted as the first prospective observational study to describe the use of IVT-AFL in treatment-naïve and previously treated patients with DME in routine clinical practice in France.

In APOLLON, treatment-naïve patients achieved a significant gain in BCVA of + 6.5 letters after 24 months of treatment with IVT-AFL (baseline, 63.8 letters; *p* < 0.001). Previously treated patients also experienced a gain in BCVA; although this gain was not statistically significant (+ 1.6 letters from a baseline of 60.5 letters; p = 0.415), this outcome indicated that overall, prior BCVA gains in these patients were maintained. More treatment-naïve patients (61.1%) than previously treated patients (41.9%) achieved a mean BCVA of ≥ 70 letters. Over 24 months, patients received a mean of 11.6 IVT-AFL injections, with a mean of 7.6 IVT-AFL injections received over the first 12 months of treatment.

Although it is important to note that the 24-month population was not identical to the 12-month population, the trends here are consistent with those of the 12-month analysis of APOLLON^13^. However, the visual acuity gain at 24 months was inferior to that at 12 months in both treatment-naïve patients (+ 6.5 letters at 24 months versus + 7.8 letters at 12 months) and previously treated patients (+ 1.6 letters at 24 months versus + 5.0 letters at 12 months). The treatment-naïve patients and previously treated patients had a similar baseline BCVA at the start of the APOLLON study and both groups received a similar mean number of injections over 12 months^13^ and 24 months. One possible reason for previously treated patients having a lower mean change in BCVA by 24 months compared with the treatment-naïve patients is that long-lasting DME may lead to chronic edema and the accumulation of structural changes in the retina that contribute to reduced visual function^16^. Secondly, this difference may have been driven by the higher proportion of patients who gained letters in the treatment-naïve cohort compared with those in the previously treated cohort (Supplementary Fig. 2).

Notably, treatment-naïve and previously treated patients had a similar CRT at baseline (441 and 453 μm), and IVT-AFL treatment markedly reduced the CRT in both subgroups (− 134 and − 130 μm from baseline). Further, the two treatment groups contained similar proportions of patients with intraretinal and subretinal fluid visible at baseline and Month 24. Thus, IVT-AFL treatment had an appreciable, positive impact on macular edema in both treatment-naïve and previously treated patients.

Overall, the outcomes of APOLLON are consistent with previously published data on the effectiveness of IVT-AFL in DME treatment^5,9–11^. The safety profile of IVT-AFL was also consistent with previous studies and no new safety findings were reported.

One of the most striking observations in this study was the proportion of patients in France who were not followed up for more than 1 year: of the 377 patients in the FAS, 77% were followed up for at least the first year, whereas less than half (45%) were followed up for 2 years or more. Low persistence and adherence have been reported in other real-world studies of IVT anti-VEGF agents, and these factors are associated with inferior visual gains^7,8^. Generally, persistence appears to be poorer in patients with DME compared with neovascular age-related macular degeneration, possibly due to complex comorbidities and a greater proportion of working-age individuals with DME^7,17^. Further, patients may rely on the superior visual acuity of their fellow eye where it is the better-seeing eye. Those patients who were followed up for the full 2 years in APOLLON had an average of approximately 20 follow-up visits, indicating the established presence of routine monthly monitoring in clinical practice in France.

Limitations of this study are inherent in the observational design, and the results for effectiveness variables should be interpreted carefully in the context of the uncontrolled setting. The time periods between follow-up visits are much more variable in real-world studies than in controlled clinical trials, in which a fixed visit schedule is maintained. Further, ophthalmologic assessments were performed according to routine clinical practice in each center; as a consequence, BCVA and CRT were not assessed at each visit, and this led to some missing data that could limit interpretation of the results. However, without patient exclusion criteria based on age, baseline BCVA, or comorbidities (as are implemented in interventional studies), observational studies such as APOLLON tend to better reflect the variety of patients with DME seen in the clinic.

## Conclusions

This final analysis of the APOLLON study showed that IVT-AFL treatment of patients with DME was associated with significant and durable improvements in visual acuity in treatment-naïve patients over 24 months, and previously treated patients maintained their initial visual acuity over the study period. Anatomic improvements achieved by 12 months were maintained in both cohorts over 24 months. Although less than half of all patients persisted with treatment over the full 24 months, patients who did persist received regular treatment and monitoring in line with the recommended treatment regimen. The safety profile of IVT-AFL was consistent with previous studies.

## Supplementary Information

Supplementary Information.

## Data Availability

Availability of the data underlying this publication will be determined according to Bayer’s commitment to the EFPIA/PhRMA “Principles for responsible clinical trial data sharing.” This pertains to scope, time point, and process of data access. As such, Bayer commits to sharing, upon request from qualified scientific and medical researchers, patient-level clinical trial data, study-level clinical trial data, and protocols from clinical trials in patients for medicines and indications approved in the United States (US) and European Union (EU) as necessary for conducting legitimate research. This applies to data on new medicines and indications that have been approved by the EU and US regulatory agencies on or after January 1, 2014. Interested researchers can use www.clinicalstudydatarequest.com to request access to anonymized patient-level data and supporting documents from clinical studies to conduct further research that can help advance medical science or improve patient care. Information on the Bayer criteria for listing studies and other relevant information is provided in the ‘Study sponsors’ section of the portal. Data access will be granted to anonymized patient-level data, protocols, and clinical study reports after approval by an independent scientific review panel. Bayer is not involved in the decisions made by the independent review panel. Bayer will take all necessary measures to ensure that patient privacy is safeguarded.
