# Supplementary material for: Two-year outcomes of the APOLLON observational study of intravitreal aflibercept monotherapy in France in patients with diabetic macular edema
Source: Sci Rep. 2022 Oct 29;12:18242. doi: 10.1038/s41598-022-22838-1 (PMC9617874; doi:10.1038/s41598-022-22838-1)

**SUPPLEMENTARY INFORMATION**

# Two-year outcomes of the APOLLON observational study of intravitreal aflibercept monotherapy in France in patients with diabetic macular edema

Jean-François Korobelnik,[1,2]* Vincent Daien,[3,4] Céline Faure,[5] Ramin Tadayoni,[6] Audrey Giocanti-Aurégan,[7] Corinne Dot,[8,9] Laurent Kodjikian,[10] Pascale Massin,[11#] on behalf of the APOLLON study investigators

[1]CHU de Bordeaux, Université de Bordeaux, France

[2]INSERM, Bordeaux Population Health Research Center, UMR1219, Université de Bordeaux, Bordeaux, France

[3]Hôpital Gui De Chauliac, Montpellier, France

[4]INSERM, Université de Montpellier, Montpellier, France

[5]Hôpital Privé Saint Martin, Ramsay Générale de Santé, Caen, France

[6]Hôpital Lariboisière, Université de Paris, Hôpital i, AP-HP, Hôpital Fondation Rothschild, Paris, France

[7]Avicenne, AP-HP, Université Paris 13, DHU Vision et Handicaps, Bobigny, France

[8]HIA Desgenettes, Lyon, France

[9]École du Val de Grâce, Paris, France

[10]Department of Ophthalmology, Croix-Rousse University Hospital, Hospices Civils de Lyon, Lyon, France; UMR-CNRS 5510 Matéis, University of Lyon, Villeurbanne, France

[11]CUDC, Hôpital Lariboisière, Paris, France

[#]Pascale Massin is now at Centre d'Ophtalmologie Paris, Breteuil, France

***Corresponding author:**

Jean-François Korobelnik

jean-francois.korobelnik@chu-bordeaux.fr

Service d'Ophtalmologie

Hôpital Pellegrin

CHU de Bordeaux

Place Amélie Raba Léon

33000 Bordeaux

France

**Supplementary Figure 1** Numbers of patients with BCVA and CRT data available at each key time point, both for the overall FAS and stratified according to treatment status. BCVA, best-corrected visual acuity; CRT, central retinal thickness; FAS, full set analysis.

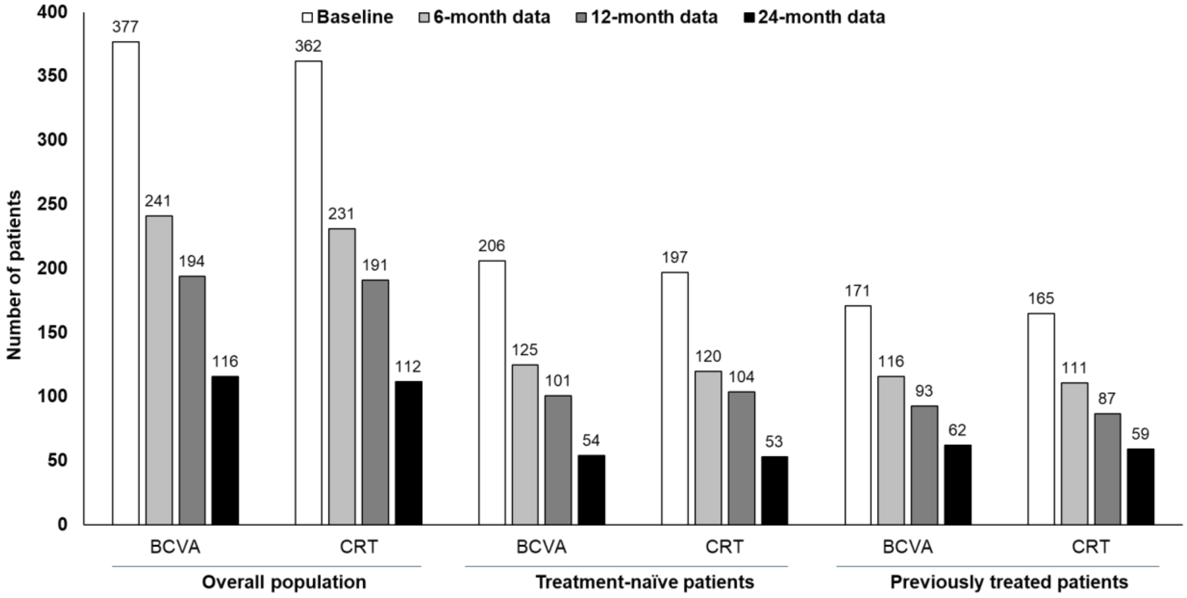


**Supplementary Figure 2** Letter gains and losses for each treatment cohort in the full analysis set by Month 24.

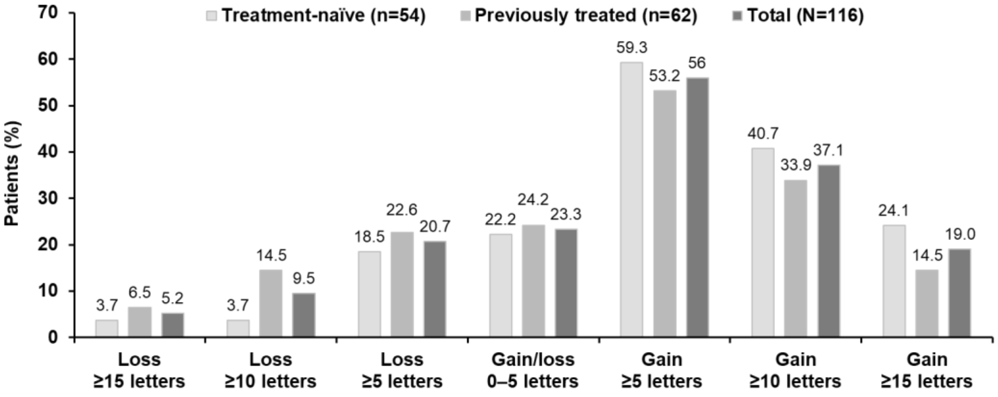

**Supplementary Figure 3** Mean BCVA letter score at baseline and Month 24 stratified by treatment cohort and baseline BCVA letter score. BCVA, best-corrected visual acuity; ETDRS, Early Treatment Diabetic Retinopathy Study.

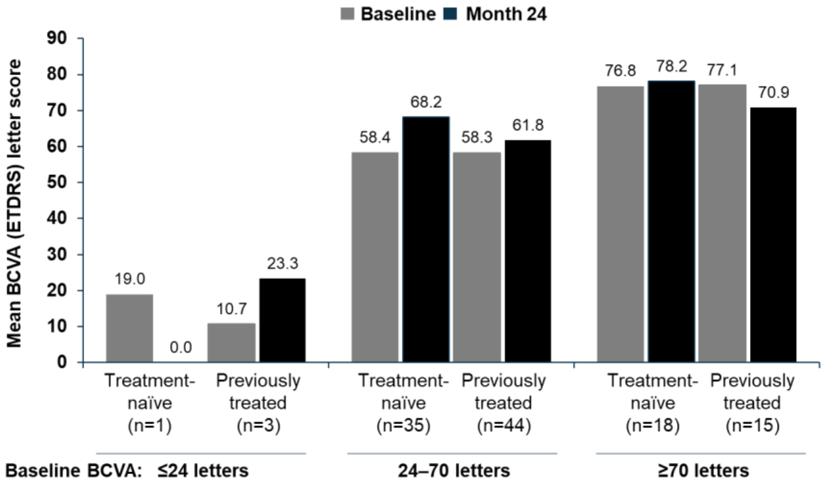

Supplement: Supplementary file 1 — Supplementary Information. [file 41598_2022_22838_MOESM1_ESM.pdf]
